# Supplementary material for: Effect of Xpert MTB/RIF testing introduction and favorable outcome predictors for tuberculosis treatment among HIV infected adults in rural southern Mozambique. A retrospective cohort study
Source: PLoS One. 2020 Mar 9;15(3):e0229995. doi: 10.1371/journal.pone.0229995 (PMC7062249; doi:10.1371/journal.pone.0229995)
Supplement: Attachment — Submitted filename: Response to reviewers.docx [file pone.0229995.s002.docx]

February 17^th^, 2020

PLOS ONE

Editor in Chief / Editorial Office

Dear Editors:

We appreciate the time and energy the reviewers put into providing comments for improvement of our manuscript. We have done our best to respond to each of their comments. Below you will find a point by point response (in italics) to the reviewer’s comments (in bold). Thank you.

Reviewer #1

1. **The manuscript by Nacarapa et. al, describes the use of Xpert MTB/RIF technology in tuberculosis diagnosis in a resource-limited, high-burden TB/HIV coinfection settings. The authors used a large sample size over a long time for this study. This manuscript addresses an important and timely topic regarding clinical TB screening before initiating ART in TB/HIV co-infection cases. This additional information can be useful in designing more effective treatment strategy for this particular risk group.**

**The authors have described some positive factors associated with a favorable TB treatment. It would be important to know, why and how these factors are linked to favorable TB treatment. I think the authors should critically discuss such aspects along with suitable literature references to support their views. This would also tremendously increase the value of the manuscript.**

*Our response: We appreciate the reviewer’s comment and agree. We have added language to address this in the Discussion section, paragraphs 3 and 4, which now read as follows:*

*“Factors that were positively associated with a favorable TB treatment outcome included female gender, having bacteriologically confirmed PTB, being HIV-negative [21,22], and a more recent year of treatment initiation. The difference in treatment outcomes between female and male patients* *has previously been described [23] but in this case is likely to be compounded by immigration to work in South Africa´s mining sector. Male miners come home for treatment when they get sick, but as soon as they feel better, they return to their work, very often without requesting a transfer or communicating the travel to the health staff. Furthermore, better outcomes in more recent years are most likely due to the improvements seen in the delivery of supportive services to persons with TB as these health programs evolved over time, for example improvements in patient follow up by community workers.[24]*

*Distance from home to clinic has been reported by different authors as a factor influencing adherence and loss to follow up in TB patients, and hence worsening treatment outcomes.[25,26] Transport cost and physical inability to walk long distances to the hospital to pick up the treatment are probably the most influential reasons leading to treatment default.”*

Reviewer #2

1. **The title of the paper does not justify the aim of the study and should be modified accordingly.**

*Our response: We appreciate the reviewer’s comments on the title. We added and adjusted the language in the title to better reflect the aim of the study. Title now reads as follows:*

*“Effect of Xpert MTB/RIF testing introduction and favorable outcome predictors for tuberculosis treatment among HIV infected adults in rural southern Mozambique. A retrospective cohort study.*

*Short title: Xpert MTB/RIF testing and tuberculosis treatment in HIV infected adults in rural Mozambique”*

2. **The authors should include information about rate of mortality in TB patients and TB- HIV co-infected patients before and after introduction of genexpert (Year-wise data would be better).**

*Our response: We appreciate the reviewer’s comment and would like to draw the attention to Table 2. Characteristics of TB patients by year, at Carmelo Hospital between January 1, 2006 and December 31, 2017. In column 8, Deaths [n (%)], information can be found by year with absolute and relative frequencies of death.*

3. **The authors should add few lines explaining the scheme of treatment followed in TB and TB-HIV co-infected patient in their country. The authors can also add information about if any changes has been incorporated and why the changes were done and in which year they were done.**

*Our response: We again appreciate the reviewer’s comment on this subject. We have added a sub-section named Anti-TB treatment regimens under Study population and methods to address this, which reads as follows:*

*“Anti-TB treatment regimens*

*In Mozambique, ATT is standard and provided free of charge exclusively by the NTP. For new patients, treatment consists of a 2-month intensive phase with a daily combination of isoniazid (H), rifampicin (R), pyrazinamide (Z), and ethambutol (E), followed by a 4-month maintenance phase with H and R. Following WHO recommendations in 2017,[18] the category II regime for retreatment is no longer used (HRZE plus streptomycin (S) for 2 months followed by 1 month of HRZE and 5 months of HRE), and it is recommended that drug sensitivity testing is conducted in patients requiring retreatment.”*

4. **Does any government health agency/NGO monitors the treatment and diagnosis of TB? A Brief information about the policy for controlling TB in the country will provide better idea to the readers.**

*Our response: We appreciate the reviewer’s comment. Please refer to changes made to paragraph two of the introduction in which we have added information to reflect that the NTP is part of the MOH.*

*“…However, only 52% of estimated cases were diagnosed and reported to the National TB Program (NTP), which is the agency of the Ministry of Health responsible for combating TB in the country.[12]…”*

Sincerely,

__________________________

Emílio Valverde

Country Director

The Aurum Institute in Moçambique

+258 823232220

+27(0) 105901300

[E.Valverde@auruminstitute.org](mailto:E.Valverde@auruminstitute.org)
